# Sleep optimization to improve glycemic control in adults with type 1 diabetes: study protocol for a randomized controlled parallel intervention trial

**DOI:** 10.1186/s13063-022-06565-6

**Published:** 2022-08-19

**Authors:** Pamela Martyn-Nemeth, Jennifer Duffecy, Laurie Quinn, Sirimon Reutrakul, Alana D. Steffen, Larisa Burke, Margaret H. Clark Withington, Ghada Abu Irsheed, Rose Perez, Minsun Park, Adam Saleh, Dan Mihailescu, Kelly Glazer Baron

**Affiliations:** 1 Department of Biobehavioral Nursing Science, College of Nursing, University of Illinois Chicago, Chicago, IL USA; 2 Department of Psychiatry, College of Medicine, University of Illinois Chicago, Chicago, IL USA; 3 Department of Endocrinology, College of Medicine, University of Illinois Chicago, Chicago, IL USA; 4 Department of Population Health Nursing Science, College of Nursing, University of Illinois Chicago, Chicago, IL USA; 5 Office of Research Facilitation, College of Nursing, University of Illinois Chicago, Chicago, IL USA; 6 Department of Endocrinology, Cook County Health, Chicago, IL USA; 7 Division of Public Health, Department of Family and Preventive Medicine, University of Utah, Salt Lake City, UT USA

**Keywords:** Type 1 diabetes, Sleep, Glycemic control, Glycemic variability, Randomized controlled trial

## Abstract

**Background:**

Despite improvements in treatment regimens and technology, less than 20% of adults with type 1 diabetes (T1D) achieve glycemic targets. Sleep is increasingly recognized as a potentially modifiable target for improving glycemic control. Diabetes distress, poor self-management behaviors, and reduced quality of life have also been linked to sleep variability and insufficient sleep duration. A significant gap of knowledge exists regarding interventions to improve sleep and the effects of sleep optimization on glycemic control in T1D. The purpose of this study is to determine the efficacy of a T1D-specific sleep optimization intervention (Sleep-Opt) on the primary outcomes of sleep variability, sleep duration, and glycemic control (A1C); other glycemic parameters (glycemic variability, time-in-range [TIR]); diabetes distress; self-management behaviors; quality of life; and other patient-reported outcomes in adults with T1D and habitual increased sleep variability or short sleep duration.

**Methods:**

A randomized controlled parallel-arm study will be employed in 120 adults (aged 18 to 65 years) with T1D. Participants will be screened for habitual sleep variability (> 1 h/week) or insufficient sleep duration (< 6.5 h per night). Eligible subjects will be randomized to the Sleep-Opt intervention group or healthy living attention control group for 12 weeks. A 1-week run-in period is planned, with baseline measures of sleep by actigraphy (sleep variability and duration), glycemia (A1C and related glycemic measures: glycemic variability and TIR using continuous glucose monitoring), and other secondary outcomes: diabetes distress, self-management behaviors, quality of life, and additional patient-reported outcomes. Sleep-Opt is a technology-assisted behavioral sleep intervention that we recently developed that leverages the rapidly increasing public interest in sleep tracking. Our behavioral intervention employs four elements: a wearable sleep tracker, didactic content, an interactive smartphone application, and brief telephone counseling. The attention control group will participate in a healthy living information program. Baseline measures will be repeated at midpoint, program completion, and post-program (weeks 6, 12, and 24, respectively) to determine differences between the two groups and sustainability of the intervention.

**Discussion:**

A better understanding of strategies to improve sleep in persons with T1D has the potential to be an important component of diabetes.

**Trial registration:**

Clinical Trial Registration: NCT04506151.

## Background

Sleep variability and insufficient sleep duration have negative health consequences in the general population. These include changes in appetite and eating patterns [1–3], obesity [4], insulin resistance [5], increased systemic inflammation [6], metabolic syndrome [7], dysglycemia [8], risk for incident diabetes [3], depression [9], and a higher prevalence of cardiovascular disease [10]. Sleep times of less than 5 h have been associated with up to four times the mortality risk of those with greater than 5 h [11, 12]. Each hour of increased sleep variability, as measured by standard deviation (SD) of sleep duration, was associated with a 27% higher odds of metabolic syndrome in a multi-ethnic population [7]. Work commitments, family and social obligations, and general stress have also been linked with poor sleep [13, 14]. Negative health consequences of insufficient and irregular sleep may be amplified for persons with type 1 diabetes (T1D), who must cope with the added burden of managing a chronic condition [15].

Up to 40% of adults with T1D had insufficient sleep (sleep duration < 6–6.5 h/night) either by self-report or objectively assessed [16–24]. Insufficient sleep is a predictor of poor glycemic control in T1D [16, 25]. Increased insulin resistance likely plays a central role; one night of experimental sleep restriction (4 h) in seven persons with T1D was associated with decreased peripheral insulin sensitivity, compared to normal sleep duration (7.8 h) [26]. In our recent meta-analysis, adults with T1D who reported sleeping > 6 h had 0.24% lower A1C levels than those sleeping ≤ 6 h [16].

In addition to insufficient sleep, sleep variability (a potential marker of circadian misalignment) can impact glycemic control. Up to 73% of adults with T1D have sleep variability (> 1 h) [17, 27]. The circadian system plays an important role in glucose metabolism, and experimental circadian misalignment results in impaired glucose tolerance [28, 29]. Thus, sleep variability could be detrimental to glycemic control. Supporting this hypothesis, recent studies have reported that sleep variability is an independent predictor of glycemic control in T1D [17, 30, 31]. Sleep variability (SD of sleep duration as objectively measured by actigraphy) explained 8.2% to 15.8% of the variance in glycemic control [17, 30]. In our study of 41 working-age adults with T1D, those with SD of sleep duration > 1 h had significantly higher A1C than those with SD sleep duration ≤ 1 h (median 7.2% vs. 7.8%, *p* = 0.008). Sleep variability was also associated with increased daily insulin requirement, suggesting more insulin resistance in these individuals [17]. These findings were reproducible: another study in 65 adolescents with T1D also found that greater SD of sleep duration was significantly associated with higher A1C [30], and in a study of 191 German adolescents with T1D, greater variability of sleep timing between work and free days was associated with higher insulin requirements [32]. Persons with T1D lack endogenous insulin secretion; varying degrees of insulin resistance could lead to increased glycemic variability (within-day glucose fluctuations), a factor reported to be associated with increased microvascular complications and cardiovascular events in T1D [33, 34]. Indeed, our pilot data in 30 adults with T1D revealed that greater SD of sleep duration was associated with greater glycemic variability as measured by continuous glucose monitoring (CGM) [27].

These data strongly suggest that sleep variability and insufficient sleep duration affect glycemic control and glycemic variability, with the effect size similar to some standard treatments for T1D [35, 36]. Despite recognition that sleep patterns should be assessed in individuals with diabetes [37], few studies have been conducted to evaluate strategies to improve sleep. Those conducted have primarily evaluated interventions in children [38, 39]. Perfect et al. conducted a short-term pilot RCT using sleep extension in 79 adolescents with T1D for 1 week [40]. The sleep extension intervention included didactic information on topics such as the importance of sleep, sleep hygiene principles, control of environmental conditions, management of competing activities, and stress reduction, as well as use of a sleep log and actigraphy monitoring [40]. The preliminary results revealed that glucose levels as measured by CGM in extension participants differed from the fixed-sleep-duration group by 17 mg/dl points (*p* = .003) during the sleep modification week. Sleep extension resulted in 11 h more spent in the glucose target range than those in the fixed-sleep condition [41]. In a pilot trial of 39 children, aged 5–9, and their parents, sleep-promoting intervention (relaxation and mindfulness, setting a bedtime, and combating bedtime resistance/nighttime waking) was compared with usual care [38]. The program was well accepted, but there was no difference in children’s total sleep time, sleep efficiency, or A1C at 3 months. However, when excluding children with A1C < 7% at baseline, there was a possible small effect of sleep coach vs. usual care on A1C (~0.3%). A similar intervention was compared to usual care in 39 adolescents with T1D [39]. The study showed excellent feasibility, and teens in the sleep coach group had an increase in sleep duration by 48 min and were less likely to report poor sleep quality compared with control group. However, no change in A1C was observed. This emerging evidence supports the feasibility and efficacy of sleep optimization, as well as a possible dose-response relationship between optimized sleep and changes in metabolic control.

Because sleep is linked to possible mediators of glycemic control (including diabetes distress [42–44], diabetes self-management behavior [45, 46], and quality of life [QoL] [47]), those mediators’ influence on glycemic control during sleep optimization need to be examined also. Diabetes distress pertains to the emotional burdens and worries associated with the complexities of managing diabetes [48]. Moderate to high distress levels are experienced by up to 54% of those with T1D [48]. Concern over blood glucose levels (particularly fear of hypoglycemia) is a major source of distress at night that impacts sleep [49]. In a study of 267 adults with T1D, diabetes distress was found to be significantly higher in those adults who reported poor sleep quality. Those with poor sleep quality also experienced greater daytime sleepiness and diabetes regimen burdens [15].

Poor sleep has also been linked directly to self-management behavior. In a cross-sectional study of 45 adolescents, a significant relationship was found between sleep duration and self-management behavior [50]. Specifically, a 15- and 20-min increase in sleep was associated with one additional blood glucose check and one additional insulin bolus, respectively [50]. In addition, sleep variability (SD of sleep duration) was found to be a significant predictor of self-management behavior, explaining 6.1% of the variance in the frequency of blood glucose monitoring [30]. Thus, improving sleep variability and duration could potentially improve self-management behavior.

In summary, we found no published studies that explored the effects of sleep optimization (strategies to improve sleep duration and variability) on glycemic control in adults with T1D. These data are needed and could have a large clinical impact, given the current state of suboptimal glycemic control and increasing incidence of T1D. Wearable sleep trackers provide a critical opportunity to engage short or variable sleepers. Over the past few years, the public’s interest in monitoring sleep has increased immensely, providing an important opportunity to affect sleep in public health. Our intervention uses data from a wearable sleep tracker (Fitbit) to personalize feedback and promote interaction with remote coaches.

Enhancing adherence to technology-assisted behavioral interventions is key to improvements. Many technology interventions suffer from high rates of non-adherence [51]. Coached interventions typically show larger effect sizes than unguided interventions, likely due to improved adherence [52]. The process by which human support enhances adherence to behavioral intervention technologies has been termed “Supportive Accountability” [53] and draws on broad empirical literature, including clinical and organizational psychology [54, 55] and motivation theory [56, 57]. Accountability is defined as knowing that one will have to justify use or non-use to another individual at some future time [54]. The model involves qualities of the coach, including legitimacy, trustworthiness, and helpfulness. We designed and tested a coaching protocol around these principles (Duffey, Kinsingre, Ludman & Mohr, Brief Telephone Support Program to Enhance Adherence to Technology Assisted Behavioral Interventions Therapist Manual, unpublished protocol) that demonstrated the capacity to enhance adherence in a sleep extension intervention.

## Methods

### Objectives

**Fig. 1:**
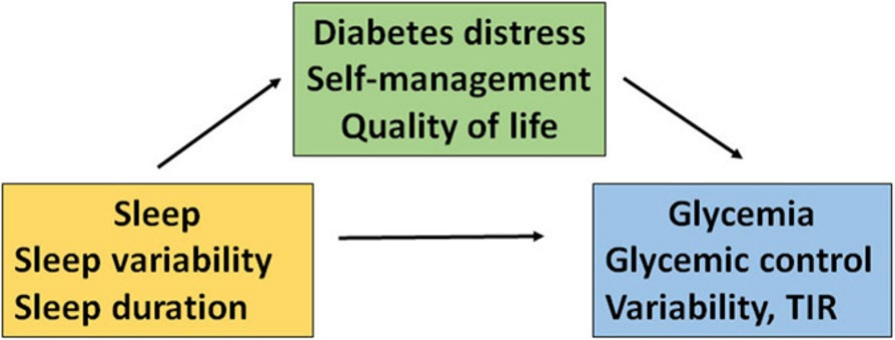
Model of relationships among sleep, glycemic control, distress, self-management behavior and QoL

The goal of this study is to improve glycemic control (A1C) by reducing sleep variability and improving insufficient sleep duration. The specific aims are:

1. Determine the effect of the Sleep-Opt intervention (compared to an attention control group) on sleep variability, sleep duration, and glycemic control (primary outcomes)
2. Determine if Sleep-Opt will result in improved psychological and behavioral outcomes, including diabetes distress, diabetes self-management behavior, QoL, fatigue, mood, and subjective sleep quality compared to the healthy living attention control group
3. Determine the contribution of changes in sleep variability and sleep duration during the intervention to changes in glycemic parameters (A1C, glycemic variability, TIR). We hypothesize that Sleep-Opt will result in improved sleep and glycemic control, lower diabetes distress, and improve self-management behavior and QoL. Reduction in variability and improved sleep duration will correlate with improvement in glycemic parameters (Fig. 1)

### Design

A randomized controlled parallel-arm design will be used. Following a baseline run-in phase, 120 subjects will be randomized to the Sleep-Opt or healthy living attention control group for 12 weeks.

### Setting

The study will be conducted remotely with participants in their free-living environment living in the United States. Due to changes instituted with COVID-19, data collection was converted to remote collection using mail services and videoconferencing.

### Recruitment

Participants will be recruited through two Midwestern medical centers, diabetes clinics, diabetes websites, and organizations, using flyers, e-announcements, recruitment letters, listservs, and ResearchMatch (www.researchmatch.org).

### Participant eligibility criteria

Inclusion criteria consist of adults 18–65 years old with a clinical diagnosis of T1D for at least 1 year who report habitual sleep variability (1 h/week or more) or sleep duration < 6.5 h/night during work- or weekdays (confirmed with actigraphy) who have a desire to improve sleep and who own a smartphone compatible with Fitbit.

Exclusion criteria consist of insomnia symptoms defined as severe as assessed by the Insomnia Severity Index [58] (score ≥ 15), being at high risk for obstructive sleep apnea as assessed by the STOP Questionnaire [59], history of severe hypoglycemia (defined as hypoglycemic episodes that result in loss of consciousness within the last 6 months, seizures, or requiring emergency room visits or hospitalization), A1C > 10%, rotating shift or night shift work, use of sleep medications/aids, significant renal impairment (estimated glomerular filtration rate < 45 ml/min/1.73 m^2^), significant medical morbidities (such as congestive heart failure, cirrhosis, chronic obstructive pulmonary disease requiring oxygen, active treatment for cancer, restless leg syndrome, depression [8-item Patient Health Questionnaire PHQ-8 score greater than or equal to 10], history of stroke with neurological deficits), or breast feeding, pregnant, or planning pregnancy.

### Consent procedures

Potentially eligible interested participants will be screened by trained study personnel for inclusion and exclusion criteria. Written informed consent will be obtained online (Research Data Capture [REDCap]) prior to performing any research procedures. The informed consent process will begin when potential subjects are contacted. The researcher will explain the study purpose, procedures, benefits, risks, confidentiality, and research subject’s rights. After all questions have been answered and the subject verbally agrees to participate, written consent will be obtained. A copy of the signed consent will be provided to the participant.

### Study procedures

Following pre-screening by phone for eligibility and informed consent, those who meet initial study criteria will be scheduled for a video conference appointment for the start of the 1-week run-in period (week 0) to obtain baseline measures, confirm eligibility (A1C, urine pregnancy [if appropriate], and actigraphy for sleep), supervise the application of a CGM device, review instructions on its care, and review instructions for completion of questionnaires using REDCap. Prior to the video conference appointment, study staff will mail study materials (pregnancy test strips, measurement tape, CGM [FreeStyle Libre Pro CGM and Reader, or Dexcom], Actiwatch [Phillips Spectrum Plus], sleep log, A1C kit, and postage-paid package to return supplies) to subjects’ home (Table 1).

**Table 1:** Study procedures

Study period											
	Enrollment	Intervention									Follow-up
Week	0	1	2	3	4	5	6	8	10	12	24
Informed consent	X										
Eligibility screen	X										
Actigraphy	X						X			X	X
CGM	X						X			X	X
A1C	X						X			X	X
Questionnaires	X						X			X	X
Randomization	X										
SleepOpt		X	X	X	X		X	X	X	X	X
Control		X	X	X	X		X	X	X	X	X
Post-program evaluation										X	

#### Randomization, allocation, and masking

Following the 1-week run-in period and a confirmation of objective sleep criteria (variability [≥ 1 h] or mean sleep duration [< 6.5 h/night] during work- or weekdays), A1C results, and other inclusion criteria, participants will be randomly assigned to the Sleep-Opt intervention or healthy living attention control group. We will use permuted blocks of 4, arranged in random order and stratified by sex, A1C (cats), and age (cats). The randomization model will be developed by the study statistician and executed through the REDCap data management system. Although the statistician created the allocation schedule for two groups, they are not aware of group identifiers and will remain blinded until after main effect models are finalized.

All study staff are unaware of future treatment allocations through restrictions in REDCap permissions. Only study staff members who are designated to obtain the allocation assignment have access to this function and do this only when an eligible participant is ready for randomization. They will then communicate the assignment to the respective interventionist (coach). All investigators and study staff who collect or have access to outcome data are masked to participant allocation. If needed, the project director would be able to unmask allocation assignment and communicate allocation assignment to the appropriate personnel.

### Sleep-opt intervention

The goal of the intervention will be to decrease sleep variability by at least 30 min and/or increase time in bed by at least 30 min. The intervention will take place over 12 weeks and be conducted remotely by phone call/video conference (Webex) at participants’ preference. Participants who are randomized to Sleep-Opt will receive the following four components: (1) a wearable sleep tracker; (2) a smartphone application with interactive feedback and tools; (3) didactic content via email lessons, reminders, and notifications; and (4) brief telephone coaching. The components are described below.

#### Wearable sleep tracker

Participants assigned to the sleep intervention will receive a Fitbit wearable sleep tracker to allow them to track their sleep and share results with the coach. Data support that consumer sleep trackers provide an estimation of sleep but are less precise than validated actigraphy devices [60, 61]. Therefore, sleep sufficiency will be measured with actigraphy, which is validated but does not currently provide real-time feedback to the wearer [62]. Fitbit data will be used in coaching sessions and for providing weekly reports.

#### Smartphone application

We will use a commercial sleep tracking application to provide participants feedback on their sleep behaviors. Participants will download the Fitbit smartphone application on their smartphone and participate in a brief training in the intervention orientation session. Participants will be trained to review and edit their Fitbit sleep log each day, thus increasing the validity of the data. Although the Fitbit application has developed the ability to enter sleep goals, these features will not be set on participants’ applications. In addition, participants will be able to use other features (e.g., step goals) but not trained or instructed on the use of these application features as part of the intervention.

#### Intervention content

Participants will receive automated content including didactic lessons for 8 of the 12 weeks, with gap weeks included beginning at week 5 for participants to work on behavior change (Table 2). The intervention content was developed by members of the team with advanced training in sleep and behavior change and has been piloted in initial user testing. The eight didactic lessons (estimated duration 8–10 min) of written and video didactic content will be delivered via email using REDCap and can be viewed on smartphone, desktop, or tablet. Content from the lessons will be reinforced in the telephone coaching sessions.

**Table 2:** Intervention didactic content and coaching schedule

Week	Content: Sleep-Opt	Content: healthy living	Coaching
1	Intro: Basics of Sleep	Introduction to healthy living; dental health	20-min engagement session
2	How to Beat Bedtime Procrastination	Handwashing	5–10 min call
3	Sleep and Type I Diabetes	Preventing infection	5–10 min call
4	Dealing with Weekends and Challenges	Body alignment and stretching	5–10 min call
5	Gap week for skill building		
6	Stress and Sleep	Lung and heart health	5–10 min call
7	Gap week for skill building		
8	The Sleep Environment	Health risks of smoking	5–10 min call
9	Gap week for skill building		
10	Effects of Sleep	Vaccination	5–10 min call
11	Gap week for skill building		
12	Maintaining Your Gains	Cancer screening	Wrap-up session

#### Coaching

All participants will be assigned to an interventionist who will be a sleep coach to monitor their progress during the study and provide telephone coaching sessions related to their sleep-related goals. The coaches will establish *legitimacy* by their knowledge of sleep and basic counseling principles. They will establish *goals* with the participants based on the participants’ values and beliefs, including the sleep-related goals and usage goals (e.g., number of days wearing the sleep tracker). *Performance monitoring* will be completed through an online dashboard visible to the coaches. The first coaching session will be a 20-min engagement session, which includes introductions, rationale for the program, clarifying roles of the coach, and the participants’ goals for the program. Coaches will provide feedback to the participants based on wearable sleep tracker data. For subsequent coaching sessions, the coach and participant will also have weekly brief (5–10 min) follow-up support calls to troubleshoot any problems with the application or wearable sleep tracker, review progress, problem solve barriers to progress, and set goals. Between sessions, the coaches will be available (mostly via email) to troubleshoot any problems with the application or wearable sleep tracker. All coaching sessions, text, and email communication will be recorded, and a selection of sessions will be coded for intervention fidelity.

### Healthy living attention control

The design of the 12-week control group is intended to control for the coach contact in the intervention group, so that we can test the intervention-related components contained in Sleep-Opt. Participants assigned to the healthy living control group will be provided eight scheduled emails with health content (e.g., dental health, handwashing, stretching exercises; 8–10 min in length) with content written at the 4th grade level or below. Participants will receive eight brief (5–10 min) telephone contact from the coach (see schedule Table 2) to determine if they received the information and if they had any questions about the materials. The schedule will mirror that of the intervention group. Coaches will not provide counseling or goal setting but may clarify terms or concepts. Participants in the healthy living control group will be instructed not to change their sleep behavior. They will be eligible to receive the sleep intervention at the end of the study. After completion of the study, they will receive a Fitbit wearable fitness tracker as part of study compensation.

**Table 3:** Measures

Variables	Measures	Frequency
Demographic and health information, caffeine, and sleep aid use	Demographic, health questionnaire, hypoglycemia unawareness, Clark Scale [63], menopausal status (STRAW+10 in women≥40 years) [64], caffeine, sleep aid use	Week 0
Primary measures: Objective sleep indices Glycemic indices Glycemic control Glycemic variability	Sleep duration, sleep variability, sleep and wake timing (Respironics Actiwatch Spectrum Plus®). Confirmed with sleep diary (bedtime, disruptions, wake time). A1C CGM (Abbott Libre® or Dexcom®): glucose variability, coefficient of variation (CV%), time-in-range [65, 66]	Weeks 0, 6, 12, 24
Secondary measures:		Weeks 0, 6, 12, 24
Diabetes distress Diabetes self-management Quality of life Fatigue Depressed mood Subjective sleep quality	T1D Diabetes Distress Scale [67] Self-Management Questionnaire-R [68] Diabetes Quality of Life Scale (DQOL) [69] PROMIS Fatigue Scale [70] Center for Epidemiological Studies Depression Scale (CES-D) [71] Pittsburgh Sleep Quality Index [72]	
Important patient-related variables	Self-Efficacy for Diabetes Scale [73], General Anxiety Disorder – 7-item (GAD-7) [74], Hypoglycemia Fear Scale II [75], Epworth Sleepiness Scale [76], activity counts	Weeks 0, 6, 12, 24
Participant engagement	Number of sessions attended, length of coaching sessions, lessons viewed, Fitbit usage	Weeks 1–12

### Measures

Measures will be obtained at baseline (week 0), midpoint of intervention (week 6), end of intervention (week 12), and post-program (week 24; Table 3).

#### Primary outcomes

- Sleep variability
- Sleep duration
- Glycemic control: hemoglobin A1C

#### Secondary outcomes

- Diabetes distress
- Diabetes self-management
- Quality of life (QoL)
- Fatigue
- Depressive mood
- Subjective sleep quality

### Glycemic assessment

1. Glycemic control will be assessed using hemoglobin A1C blood spot (A1C; Home Access Health Corp.). A1C is a gold standard marker of glycemic control in T1D, reflecting average glucose levels in the previous 90 days
2. Glycemic variability using CGM will be conducted using FreeStyle Libre Pro glucose sensor or Dexcom (FDA-approved). The system captures interstitial glucose and records the data every 5–15 min. Variables to be derived from the CGM are mean glucose level, standard deviation (SD), coefficient of variation (CV), percentage of time spent in range (70–180 mg/dL), percentage of time < 70 mg/dl, and percentage of time ≥ 180 mg/dl [65, 66]. Interstitial glucose measurements with FreeStyle Libre and Dexcom were found to be accurate compared with capillary blood glucose reference values, with a mean absolute relative difference (MARD) of 12% and 9% (respectively) compared to the gold standard YSI measure of blood glucose [77]. Accuracy of 10% MARD has been approved for self-adjustment of insulin doses in clinical practice [78].

### Sleep assessment

Participants will wear an Actiwatch Spectrum Plus (Respironics, USA) on their non-dominant wrist for 1 week for assessment at baseline and weeks 6, 12, and 24. Data will be collected in 30-s epochs. Subjects will be asked to keep a daily sleep log and press an event marker on the Actiwatch at bedtime and wake-up time. Data will be downloaded and reviewed with each participant to clarify inconsistencies when the Actiwatch is returned. Bedtime and wake time will be set by researchers considering event markers, times on sleep logs, light, and activity signals as previously described [79]. Using the Immobile Minutes algorithm in the Actiware 6 software, we will derive the following variables: sleep onset, sleep offset, sleep duration, mid-sleep time (time point between sleep onset and wake time), and SD of sleep duration, an indicator of sleep variability which we previously showed to be related to glucose metabolism [17].

### Secondary outcomes: diabetes distress, self-management behavior, quality of life, fatigue, depressive mood, subjective sleep quality

Diabetes distress will be measured with the Type 1 Diabetes Distress Scale [67]. This 28-item, 6-point Likert scale measures seven subscales (powerlessness, management distress, hypoglycemia distress, negative social perceptions, eating distress, physician distress, and friend/family distress) and provides an overall total distress scale score.

Self-management behavior will be measured with the Diabetes Self-Management Questionnaire-Revised (DSMQ-R) [68]. This 27-item, 4-point Likert scale measures aspects of self-management behavior and has questions that are specific to those using rapid-acting insulin.

Quality of life will be measured with the Diabetes Quality of Life Scale (DQOL), a 46-item, 5-point Likert scale that measures four subscales (satisfaction, impact, social/vocational worry, and diabetes-related worry) [69]. The scales chosen have strong psychometric properties and have been validated in people with T1D.

Fatigue will be measured with the PROMIS Short Form 8a Fatigue Scale [70]. This 8-item, 5-point Likert scale measures the level of fatigue over the past 7 days. It uses item-response theory and has been validated for use across all populations.

Depressive mood will be measured with the Center for Epidemiological Studies Depression Scale (CES-D) [71]. This 20-item, 4-point Likert scale measures emotions over the past week. Scores range from 0 to 60. The scale has been validated in adult populations. A score ≥ 16 indicates a depressive mood.

Subjective sleep quality will be measured with the Pittsburgh Sleep Quality Index (PSQI) [72]. The PSQI measures seven domains of sleep—quality, latency, duration, efficiency, disturbances, use of sleep medications, and daytime dysfunction—over the past month. The scale provides an overall summary score. Scores of 5 or more indicate poor overall sleep quality. The scale has been psychometrically validated in a variety of adult populations, including those with diabetes.

### Additional important patient-related variables

Self-efficacy, anxiety, fear of hypoglycemia, and daytime sleepiness will be measured with validated instruments: Self-Efficacy for Diabetes Scale [80], General Anxiety Disorder – 7-item (GAD-7) [74], Hypoglycemia Fear Scale II [75], and Epworth Sleepiness Scale [76]. Because menopausal status can affect sleep, we will use the STRAW+10 (Stages of Reproductive Aging Workshop+10) criteria for staging menopause for women aged 40 and over [64]. Physical activity will be obtained by activity counts from actigraphy recordings.

### Sample size calculation

We conservatively estimated the minimal detectable difference between treatment arms to be 0.4 to 0.6 standard deviations for our target sample size of 60 per group (after attrition) at the 12-week post-treatment measurement based on a two-groups pre-post design, *α* = 0.02, two-sided, 80% power, and assuming correlations between time points of 0.5 to 0.8 [81]. Standard deviations from pilot data for A1C (1.07%), sleep variability (30 min), and sleep duration (49 min), and the correlation between measurements (*r* = 0.56 to 0.85) were used for sample size determination. Our minimal detectable difference represents a modest but clinically important change (e.g., 20-min increase in sleep duration, 0.43% change in A1C).

### Data analysis

Aim 1: Determine the effect of Sleep-Opt (compared to a healthy living attention control group) on the primary outcomes of sleep variability, sleep duration, and glycemic control (A1C).

We will conduct mixed-effect models for repeated measures (MMRM) using change from baseline for our outcome regressed onto categorical fixed effects for treatment arm, time, their interaction, and the initial baseline measure of the outcome. We will use an unstructured covariance structure to model within-person errors. If convergence problems occur, we will select the best fitting model from among several options, including random coefficients with residual covariance patterns such as autoregressive or exchangeable structure [82, 83]. In addition to A1C, we will estimate separate models for parameters from CGM (glycemic variability, TIR). Sex will be included as a covariate and tested for moderation of the treatment effect. We will also control for BMI, A1C (for other glucose measures), and method of insulin delivery. If treatment arms are found to differ in the distribution of baseline characteristics despite randomization, we will conduct sensitivity analyses including these variables as covariates. The primary endpoint will be change differences between groups at 12 weeks, based on least square means using a two-sided test with *α* = .05. We will also assess differences in change from baseline to the 24-week endpoint to assess sustainability of effect.

Aim 2: Determine if Sleep-Opt will result in improved psychological and behavioral secondary outcomes, including diabetes distress, diabetes self-management behavior, and QoL. Secondary outcomes will be analyzed with the same approach used in aim 1

Aim 3: Determine the contribution of changes in sleep variability and sleep duration during the intervention to changes in glycemic parameters (A1C, glycemic variability, TIR)

Sleep-Opt is designed to reduce sleep variability and extend sleep and duration, and we expect these changes to mediate change in glycemic control. In the context of the Aim 1 models, we will add time-varying sleep parameters—considering the average levels per person and the variation at each time point—to understand contributions of between- and within-person differences. We will also examine additional covariates predicting sleep parameters, because level and change in sleep parameters (while influenced by randomized treatment arm) are not experimentally controlled [84]. In addition, we will test moderation of within-person mechanisms by sex, distress, method of insulin delivery, and A1C level using interaction terms. Successful completion of this aim will inform how aspects of sleep are related to the various aspects of glycemic control in general; which glycemic control parameters show reactivity to within-person fluctuations in sleep; and which personal characteristics may be more associated with this reactivity. This will explicate key mechanisms of change and suggest who may benefit most from sleep optimization.

### Methods to address missing data

While missing data will be minimized through careful procedures, some missing data are inevitable with longitudinal studies. We will handle missing data using the full information maximum likelihood (FIML) approach that is appropriate for data missing at random [85]. We will use inclusive models with auxiliary variables related to missingness among covariates collected at baseline, if needed, to support the missing-at-random assumption [86]. Multiple imputation will be considered if excessive data are missing among predictor variables (e.g., change in sleep parameters for Aim 3) [86]. Sensitivity analyses such as pattern mixture models will be employed if data are suspected to be missing not at random [82].

### Data safety monitoring committee composition and function, reporting of adverse events

The Data Safety Monitoring Committee (DSMC) will be an independent committee, composed of five senior faculty members whose roles include a statistician, endocrinologist, and sleep and trials specialists. The DSMC will meet annually but will be consulted more frequently if needed. An annual summary report will be provided to the principal investigator, IRB, and funding organization. Adverse and unanticipated events will be reported to the IRB and sponsor according to IRB protocol and a summary provided to the DSMC.

### Frequency and plans for monitoring trial conduct

The principal investigator (PI) will have overall responsibility for day-to-day support of the trial. A trial steering committee (SC) will be composed of all investigators and study staff (see title page for members). The SC will meet monthly and review recruitment, enrollment, retention, completion, and intervention fidelity reports.

A trial management committee, comprised of the PI, project manager and research specialist will meet weekly to monitor study plans, weekly recruitment and enrollment processes, randomization, progress of study participants, supply and equipment purchases, preparation of agenda and materials for SC and DSMC meetings.

Intervention fidelity will be evaluated quarterly by study personnel and reported to the SC. All coaching sessions (Sleep-Opt and healthy living attention control) will be recorded for training and fidelity monitoring. Approximately 10% of conducted sessions will be reviewed and coded for adherence using previously developed rating scales [87]. A manual of operations will be developed, and staff will be trained on study procedures. All interactions with participants will be scripted when possible. Fidelity less than 88% will trigger retraining.

Ten percent of actigraphy recordings will be reviewed for congruence in scoring by one of the study co-investigators who is not involved in data collection. Congruence less than 88% will trigger retraining.

### Participant retention strategy

We expect to randomize 144 subjects to obtain complete data on 120 subjects. This estimate is based on our previous work, with a 17% attrition rate expected. All efforts will be made to retain participants and reduce burden of participation. Assessment visits and coaching calls will be flexibly scheduled according to participant needs. Participants will be compensated for participation incrementally across visits. Participant sleep and glucose data will be provided at the end of the study. Participants will also be allowed to keep the Fitbit device as an additional incentive for program completion. Those in the attention control group will receive a Fitbit at the end of the study.

## Discussion

Despite improvements in treatment regimens and technology, less than 20% of adults with T1D achieve glycemic targets [88]. Sleep is increasingly recognized as a potentially modifiable target for improving glycemic control. Research is limited as to how to optimize sleep among persons with T1D and whether such interventions improve important outcomes, including glycemic control, diabetes distress, and QoL. The proposed study will determine the efficacy of a T1D-specific sleep optimization intervention (Sleep-Opt) in reducing sleep variability and insufficient sleep duration and improving glycemic control, other glucose parameters, diabetes distress, self-management, QoL, and other important patient-reported outcomes. If the intervention is determined to be beneficial, sleep optimization could be incorporated as a component of standard medical care of T1D.

### Trial status

Protocol number version 10, November 2021. The first participant was randomized on January 19, 2021. The trial will complete recruitment on April 30, 2025.

IRB Protocol #2020-0374

All protocol amendments will be submitted for approval by the IRB prior to implementation of any changes. Any changes to the study aims will require approval by the funding organization prior to implementation. Written informed consent to participate will be obtained from all participants.

## Abbreviations

T1D: Type 1 diabetes
QoL: Quality of life
SD: Standard deviation
REDCap: Research electronic data capture
CGM: Continuous glucose monitoring
DQOL: Diabetes Quality of Life Scale
CES-D: Center for Epidemiologic Studies-Depression Scale
PSQI: Pittsburgh Sleep Quality Index
PROMIS: Patient-Reported Outcomes Measurement Information System
GAD-7: Generalized Anxiety Disorder Scale-7-item
STRAW-10: Stage of Reproductive Aging+10
CV: Coefficient of variation
TIR: Time-in-range
MMRM: Mixed-effect models for repeated measures
MARD: Mean absolute relative difference
FIML: Full information maximum likelihood
DSMQ-R: Diabetes Self-Management Questionnaire-Revised
DSMC: Data Safety Monitoring Committee
SC: Trial steering committee
PI: Principle investigator

## References

[1] Spiegel K, Tasali E, Penev P, Van Cauter E (2004). Brief communication: Sleep curtailment in healthy young men is associated with decreased leptin elvels, elevated ghrelin levels, and increased hunger and appetite. Ann Intern Med.

[2] Nedeltcheva AV, Kilkus JM, Imperial J, Kasza K, Schoeller AA, Penev PD (2009). Sleep curtailment is accompanied by increased intake of calories from snacks. Am J Clin Nutr.

[3] Reutrakul S, Van Cauter E (2014). Interactions between sleep, circadian function, and glucose metabolism: implications for risk and severity of diabetes. Ann N Y Acad Sci.

[4] Cappuccio FP, Taggart FM, Kandala NB, Currie A, Peile E, Stranges S (2008). Meta-analysis of short sleep duration and obesity in children and adults. Sleep..

[5] Klingenberg L, Chaput JP, Holmback U, Visby T, Jennum P, Nikolic M (2013). Acute sleep restriction reduces insulin sensitivity in adolescent boys. Sleep..

[6] Okun ML, Reynolds CF, Buysse DJ, Monk TH, Mazumdar S, Begley A (2011). Sleep variability, health-related practices, and inflammatory markers in a community dwelling sample of older adults. Psychosom Med.

[7] Huang T, Redline S (2019). Cross-sectional and prospective associations of actigraphy-assessed sleep regularity with metabolic abnormalities: the multi-ethnic study of atherosclerosis. Diabetes Care.

[8] Zhu Y, Li AM, Au CT, Kong AP, Zhang J, Wong CK, et al. Association between sleep architecture and glucose tolerance in children and adolescents. J Diabetes. 2015;7(1):10–15. 10.1111/1753-0407.12138 25695111

[9] Meerlo P, Havekes R, Steiger A (2015). Chronically restricted or disrupted sleep as a causal factor in the development of depression. Curr Top Behav Neurosci.

[10] Cappuccio FP, Cooper D, D'Elia L, Strazzullo P, Miller MA (2011). Sleep duration predicts cardiovascular outcomes: a systematic review and meta-analysis of prospective studies. Eur Heart J.

[11] Vgontzas AN, Liao D, Pejovic S, Calhoun S, Karataraki M, Basta M (2010). Insomnia with short sleep duration and mortality: the Penn State cohort. Sleep..

[12] Kripke DF, Langer RD, Elliott JA, Klauber MR, Rex KM (2011). Mortality related to actigraphic long and short sleep. Sleep Med.

[13] Owens J (2014). Insufficient sleep in adolescents and young adults: an update on causes and consequences. Pediatrics..

[14] Winzeler K, Voellmin A, Schafer V, Meyer AH, Cajochen C, Wilhelm FH (2014). Daily stress, presleep arousal, and sleep in healthy young women: a daily life computerized sleep diary and actigraphy study. Sleep Med.

[15] Nefs G, Donga E, van Someren E, Bot M, Speight J, Pouwer F (2015). Subjective sleep impairment in adults with type 1 or type 2 diabetes: Results from Diabetes MILES--The Netherlands. Diabetes Res Clin Pract.

[16] Reutrakul S, Thakkinstian A, Anothaisintawee T, Chontong S, Borel AL, Perfect MM (2016). Sleep characteristics in type 1 diabetes and associations with glycemic control: systematic review and meta-analysis. Sleep Med.

[17] Chontong S, Saetung S, Reutrakul S (2016). Higher sleep variability is associated with poorer glycaemic control in patients with type 1 diabetes. J Sleep Res.

[18] Estrada CL, Danielson KK, Drum ML, Lipton RB (2012). Insufficient sleep in young patients with diabetes and their families. Biol Res Nurs.

[19] Bouhassira D, Letanoux M, Hartemann A (2013). Chronic pain with neuropathic characteristics in diabetic patients: a French cross-sectional study. PLoS One.

[20] Matejko B, Kiec-Wilk B, Szopa M, Morawska IT, Malecki MT, Klupa T (2015). Are late-night eating habits and sleep duration associated with glycemic control in adult type 1 diabetes patients treated with insulin pumps?. J Diabetes Invest.

[21] Denic-Roberts H, Costacou T, Orchard TJ (2016). Subjective sleep disturbances and glycemic control in adults with long-standing type 1 diabetes: The Pittsburgh's Epidemiology of Diabetes Complications study. Diabetes Res Clin Pract.

[22] Borel AL, Pepin JL, Nasse L, Baguet JP, Netter S, Benhamou PY (2013). Short sleep duration measured by wrist actimetry is associated with deteriorated glycemic control in type 1 diabetes. Diabetes Care.

[23] van Dijk M, Donga E, van Dijk JG, Lammers GJ, van Kralingen KW, Dekkers OM (2011). Disturbed subjective sleep characteristics in adult patients with long-standing type 1 diabetes mellitus. Diabetologia..

[24] Bachle C, Lange K, Stahl-Pehe A, Castillo K, Holl RW, Giani G (2015). Associations between HbA1c and depressive symptoms in young adults with early-onset type 1 diabetes. Psychoneuroendocrinology..

[25] Jaser SS, Foster NC, Nelson BA, Kittelsrud JM, DiMeglio LA, Quinn M (2017). Sleep in children with type 1 diabetes and their parents in the T1D Exchange. Sleep Med.

[26] Donga E, van Dijk M, van Dijk JG, Biermasz NR, Lammers GJ, Van Kralingen K (2010). Partial sleep restriction decreases insulin sensitivity in type 1 diabetes. Diabetes Care.

[27] Martyn-Nemeth P, Quinn L, Park C, Reutrakul S (2019). Objectively measured sleep variability is associated with glucose, diabetes distress and fear of hypoglycemia in adults with type 1 diabetes. Advanced Technologies & Treatments for Diabetes.

[28] Scheer FA, Hilton MF, Mantzoros CS, Shea SA (2009). Adverse metabolic and cardiovascular consequences of circadian misalignment. Proc Natl Acad Sci U S A.

[29] Leproult R, Holmback U, Van Cauter E (2014). Circadian misalignment augments markers of insulin resistance and inflammation, independently of sleep loss. Diabetes..

[30] Patel NJ, Savin KL, Kahanda SN, Malow BA, Williams LA, Lochbihler G, et al. Sleep habits in adolescents with type 1 diabetes: variability in sleep duration linked with glycemic control. Pediatr Diabetes. 2018;19:1100–6. 10.1111/pedi.12689 PMC6207484 29708297

[31] Larcher S, Gauchez AS, Lablanche S, Pepin JL, Benhamou PY, Borel AL (2016). Impact of sleep behavior on glycemic control in type 1 diabetes: the role of social jetlag. Eur J Endocrinol.

[32] von Schnurbein J, Boettcher C, Brandt S, Karges B, Dunstheimer D, Galler A (2018). Sleep and glycemic control in adolescents with type 1 diabetes. Pediatr Diabetes.

[33] Virk SA, Donaghue KC, Cho YH, Benitez-Aguirre P, Hing S, Pryke A (2016). Association between HbA1c variability and risk of microvascular complications in adolescents with type 1 diabetes. J Clin Endocrinol Metabol.

[34] Yoon JE, Sunwoo JS, Kim JS, Roh H, Ahn MY, Woo HY (2017). Poststroke glycemic variability increased recurrent cardiovascular events in diabetic patients. J Diabetes Complications.

[35] Yeh HC, Brown TT, Maruthur N, Ranasinghe P, Berger Z, Suh YD (2012). Comparative effectiveness and safety of methods of insulin delivery and glucose monitoring for diabetes mellitus: a systematic review and meta-analysis. Ann Intern Med.

[36] Bell KJ, Barclay AW, Petocz P, Colagiuri S, Brand-Miller JC (2014). Efficacy of carbohydrate counting in type 1 diabetes: a systematic review and meta-analysis. Lancet Diabetes Endocrinol.

[37] Draznin B, Aroda VR, Bakris G, Benson G, Brown FM, Freeman R (2022). 4. Comprehensive medical evaluation and assessment of comorbidities: Standards of Medical Care in Diabetes-2022. Diabetes Care.

[38] Jaser SS, Bergner EM, Hamburger ER, Bhatia S, Lyttle M, Bell GE (2021). Pilot trial of a sleep-promoting intervention for children with type 1 diabetes. J Pediatr Psychol.

[39] Jaser SS, Hamburger ER, Bergner EM, Williams R, Slaughter JC, Simmons JH (2020). Sleep coach intervention for teens with type 1 diabetes: Randomized pilot study. Pediatr Diabetes.

[40] Perfect MM, Beebe D, Levine-Donnerstein D, Frye SS, Bluez GP, Quan SF (2016). The development of a clinically relevant sleep modification protocol for youth with type 1 diabetes. Clin Pract Pediatr Psychol.

[41] Perfect MM, Frye SS, Bluez GP. The effects of a sleep extension intervention on glucose control in youth with type 1 diabetes. Diabetes. 2018;67(Supplement_1).

[42] Strandberg RB, Graue M, Wentzel-Larsen T, Peyrot M, Rokne B (2014). Relationships of diabetes-specific emotional distress, depression, anxiety, and overall well-being with HbA1c in adult persons with type 1 diabetes. J Psychosom Res.

[43] Sturt J, Dennick K, Due-Christensen M, McCarthy K (2015). The detection and management of diabetes distress in people with type 1 diabetes. Curr Diab Rep.

[44] Martyn-Nemeth P, Phillips SA, Mihailescu D, Farabi SS, Park C, Lipton R (2018). Poor sleep quality is associated with nocturnal glycaemic variability and fear of hypoglycaemia in adults with type 1 diabetes. J Adv Nurs.

[45] Hessler DM, Fisher L, Polonsky WH, Masharani U, Strycker LA, P. (2017). Diabetes distress is linked with worsening diabetes management over time in adults with Type 1 diabetes. Diabetic Med.

[46] Martyn-Nemeth P, Quinn L, Hacker E, Park H, Kujath AS (2014). Diabetes distress may adversely affect the eating styles of women with type 1 diabetes. Acta Diabetol.

[47] Perfect MM, Patel PG, Scott RE, Wheeler MD, Patel C, Griffin K (2012). Sleep, glucose, and daytime functioning in youth with type 1 diabetes. Sleep..

[48] Fisher L, Hessler D, Polonsky W, Strycker L, Masharani U, Peters A (2016). Diabetes distress in adults with type 1 diabetes: Prevalence, incidence and change over time. J Diabetes Complications.

[49] Martyn-Nemeth P, Schwarz Farabi S, Mihailescu D, Nemeth J, Quinn L (2016). Fear of hypoglycemia in adults with type 1 diabetes: impact of therapeutic advances and strategies for prevention - a review. J Diabetes Complications.

[50] McDonough RJ, Clements MA, DeLurgio SA, Patton SR (2017). Sleep duration and its impact on adherence in adolescents with type 1 diabetes mellitus. Pediatr Diabetes.

[51] Andersson G, Cuijpers P (2009). Internet-based and other computerized psychological treatments for adult depression: a meta-analysis. Cogn Behav Ther.

[52] Titov N, Andrews G, Davies M, McIntyre K, Robinson E, Solley K (2010). Internet treatment for depression: a randomized controlled trial comparing clinician vs. technician assistance. PLoS One.

[53] Mohr DC, Cuijpers P, Lehman K (2011). Supportive accountability: a model for providing human support to enhance adherence to eHealth interventions. J Med Internet Res.

[54] Lerner JS, Tetlock PE (1999). Accounting for the effects of accountability. Psychol Bull.

[55] Tyler TR (1997). The psychology of legitimacy: a relational perspective on voluntary deference to authorities. Pers Soc Psychol Rev.

[56] Deci EL, Koestner R, Ryan RM (1999). A meta-analytic review of experiments examining the effects of extrinsic rewards on intrinsic motivation. Psychol Bull.

[57] Miller WR, Rollnick S (2002). Motivational interviewing: prepairing people for change.

[58] Morin CM, Belleville G, Belanger L, Ivers H (2011). The Insomnia Severity Index: psychometric indicators to detect insomnia cases and evaluate treatment response. Sleep..

[59] Netzer NC, Stoohs RA, Netzer CM, Clark K, Strohl KP (1999). Using the Berlin Questionnaire to identify patients at risk for the sleep apnea syndrome. Ann Intern Med.

[60] Meltzer LJ, Hiruma LS, Avis K, Montgomery-Downs H, Valentin J (2015). Comparison of a commercial accelerometer with polysomnography and actigraphy in children and adolescents. Sleep..

[61] Montgomery-Downs HE, Insana SP, Bond JA (2012). Movement toward a novel activity monitoring device. Sleep Breath.

[62] Marino M, Li Y, Rueschman MN, Winkelman JW, Ellenbogen JM, Solet JM (2013). Measuring sleep: accuracy, sensitivity, and specificity of wrist actigraphy compared to polysomnography. Sleep..

[63] Clark, et al. Reduced Awareness of Hypoglycemia in Adults with IDDM. Diab Care. 1995;18(4):517–22. 10.2337/diacare.18.4.517 7497862

[64] Harlow SD, Gass M, Hall JE, Lobo R, Maki P, Rebar RW (2012). Executive summary of the Stages of Reproductive Aging Workshop + 10: addressing the unfinished agenda of staging reproductive aging. Menopause (New York, NY).

[65] Bergenstal RM, Ahmann AJ, Bailey T, Beck RW, Bissen J, Buckingham B (2013). Recommendations for standardizing glucose reporting and analysis to optimize clinical decision making in diabetes: the Ambulatory Glucose Profile (AGP). Diabetes Technol Ther.

[66] Rodbard D (2009). New and improved methods to characterize glycemic variability using continuous glucose monitoring. Diabetes Technol Ther.

[67] Fisher L, Polonsky WH, Hessler DM, Masharani U, Blumer I, Peters AL (2015). Understanding the sources of diabetes distress in adults with type 1 diabetes. J Diabetes Complications.

[68] Schmitt A, Gahr A, Hermanns N, Kulzer B, Huber J, Haak T (2013). The Diabetes Self-Management Questionnaire (DSMQ): development and evaluation of an instrument to assess diabetes self-care activities associated with glycaemic control. Health Qual Life Outcomes.

[69] DCCT Research Group (1988). Reliability and validity of a diabetes quality-of-life measure for the diabetes control and complications trial (DCCT). The DCCT Research Group. Diabetes Care.

[70] Cella D, Lai JS, Jensen SE, Christodoulou C, Junghaenel DU, Reeve BB (2016). PROMIS Fatigue Item Bank had clinical validity across diverse chronic conditions. J Clin Epidemiol.

[71] Radloff L (1977). The CES-D scale: a self-report depression scale for research in the general population. Appl Psychol Measur.

[72] Buysse DJ, Reynolds CF, Monk TH, Berman SR, Kupfer DJ (1989). The Pittsburgh Sleep Quality Index: a new instrument for psychiatric practice and research. Psychiatry Res.

[73] Lorig K. Self-Efficacy for Diabetes: Stanford University; Available from https://acl.gov/sites/default/files/nutrition/Appendix%20L%20Stanford%20Self-Efficacy%20for%20Diabetes%20Survey_508.pdf. Accessed 10 Aug 2022.

[74] Jordan P, Shedden-Mora MC, Lowe B (2017). Psychometric analysis of the Generalized Anxiety Disorder scale (GAD-7) in primary care using modern item response theory. PLoS One.

[75] Gonder-Frederick LA, Schmidt KM, Vajda KA, Greear ML, Singh H, Shepard JA (2011). Psychometric properties of the hypoglycemia fear survey-ii for adults with type 1 diabetes. Diabetes Care.

[76] Johns MW (1991). A new method for measuring daytime sleepiness: the Epworth sleepiness scale. Sleep..

[77] Bailey T, Bode BW, Christiansen MP, Klaff LJ, Alva S (2015). The performance and usability of a factory-calibrated flash glucose monitoring system. Diabetes Technol Ther.

[78] Kovatchev BP, Patek SD, Ortiz EA, Breton MD (2015). Assessing sensor accuracy for non-adjunct use of continuous glucose monitoring. Diabetes Technol Ther.

[79] Patel SR, Weng J, Rueschman M, Dudley KA, Loredo JS, Mossavar-Rahmani Y (2015). Reproducibility of a standardized actigraphy scoring algorithm for sleep in a US Hispanic/Latino population. Sleep..

[80] Ritter PL, Lorig K, Laurent DD (2016). Characteristics of the Spanish- and English-language self-efficacy to manage diabetes scales. Diabetes Educ.

[81] Rosner B. Fundamentals of biostatistics. 7th ed. Boston: Brooks/Cole; 2011.

[82] Hedeker D, Gibbons RD. Longitudinal data analysis: Wiley-Interscience; 2006.

[83] Mallinckrod CH, Lane PW, Schnell D, Peng Y, Mancuso JP (2008). Recommendations for the primary analysis of continuous endpoints in longitudinal clinical trials. Drug Inf J.

[84] Hayes AF. Introduction to mediation, moderation, and conditional process analysis: a regression-based approach: Guilford publications; 2017.

[85] Graham JW (2009). Missing data analysis: making it work in the real world. Annu Rev Psychol.

[86] Enders CK. Applied missing data analysis: Guilford press; 2010.

[87] Mohr DC, Duffecy J, Ho J, Kwasny M, Cai X, Burns MN (2013). A randomized controlled trial evaluating a manualized TeleCoaching protocol for improving adherence to a web-based intervention for the treatment of depression. PLoS One.

[88] Foster NC, Beck RW, Miller KM, Clements MA, Rickels MR, DiMeglio LA (2019). State of type 1 diabetes management and outcomes from the T1D exchange in 2016-2018. Diabetes Technol Ther.

